# Evaluation of bias induced by viral enrichment and random amplification protocols in metagenomic surveys of saliva DNA viruses

**DOI:** 10.1186/s40168-018-0507-3

**Published:** 2018-06-28

**Authors:** Marcos Parras-Moltó, Ana Rodríguez-Galet, Patricia Suárez-Rodríguez, Alberto López-Bueno

**Affiliations:** Centro de Biología Molecular Severo Ochoa (Universidad Autónoma de Madrid/Consejo Superior de Investigaciones Científicas), Madrid, Spain

## Abstract

**Background:**

Viruses are key players regulating microbial ecosystems. Exploration of viral assemblages is now possible thanks to the development of metagenomics, the most powerful tool available for studying viral ecology and discovering new viruses. Unfortunately, several sources of bias lead to the misrepresentation of certain viruses within metagenomics workflows, hindering the shift from merely descriptive studies towards quantitative comparisons of communities. Therefore, benchmark studies on virus enrichment and random amplification protocols are required to better understand the sources of bias.

**Results:**

We assessed the bias introduced by viral enrichment on mock assemblages composed of seven DNA viruses, and the bias from random amplification methods on human saliva DNA viromes, using qPCR and deep sequencing, respectively. While iodixanol cushions and 0.45 μm filtration preserved the original composition of nuclease-protected viral genomes, low-force centrifugation and 0.22 μm filtration removed large viruses. Comparison of unamplified and randomly amplified saliva viromes revealed that multiple displacement amplification (MDA) induced stochastic bias from picograms of DNA template. However, the type of bias shifted to systematic using 1 ng, with only a marginal influence by amplification time. Systematic bias consisted of over-amplification of small circular genomes, and under-amplification of those with extreme GC content, a negative bias that was shared with the PCR-based sequence-independent, single-primer amplification (SISPA) method. MDA based on random priming provided by a DNA primase activity slightly outperformed those based on random hexamers and SISPA, which may reflect differences in ability to handle sequences with extreme GC content. SISPA viromes showed uneven coverage profiles, with high coverage peaks in regions with low linguistic sequence complexity. Despite misrepresentation of certain viruses after random amplification, ordination plots based on dissimilarities among contig profiles showed perfect overlapping of related amplified and unamplified saliva viromes and strong separation from unrelated saliva viromes. This result suggests that random amplification bias has a minor impact on beta diversity studies.

**Conclusions:**

Benchmark analyses of mock and natural communities of viruses improve understanding and mitigate bias in metagenomics surveys. Bias induced by random amplification methods has only a minor impact on beta diversity studies of human saliva viromes.

**Electronic supplementary material:**

The online version of this article (10.1186/s40168-018-0507-3) contains supplementary material, which is available to authorized users.

## Background

Viruses are the most abundant and genetically diverse components of microbial ecosystems [1–3]. Unlike cellular organisms, viruses lack universal marker genes for assessment of whole viral assemblages, hampering our understanding of these key players of microbiota homeostasis. The incorporation of next-generation sequencing (NGS) technologies into metagenomic surveys of viruses has circumvented this limitation, triggering an exponential increase in the number of viromes available in databases [4]. Currently, metagenomics is the most powerful tool for studying viral ecology [5–11], and its application to host microbiomes has enabled the identification of many new viruses, including ones that infect humans [12–16]. However, caution must be taken when extracting ecological conclusions from metagenomic studies, because putative bias in viral representation can affect every step of sample manipulation.

Only a small percentage of the total DNA retrieved from human microbiomes corresponds to viral genomes [17–19]. Thus, a variety of physical virus-like particle (VLP) enrichment protocols has been employed to increase their relative ratio, enabling deep exploration of viral assemblages [17, 18, 20–25]. Most of these protocols combine low-speed centrifugation, 0.22–0.45 μm filtration, or ultracentrifugation in density gradients to remove cellular contamination and concentrate VLPs, with nuclease treatment for elimination of unprotected genetic material. Unfortunately, viruses encompass a wide range of sizes, morphologies and chemical constituents. These features endow viruses with different resistance levels to chemical and mechanical stressors, making the establishment of a universal protocol for viral genomic purification unfeasible. For example, CsCl density gradients are frequently used for efficient removal of cellular contamination during preparation of viromes [17, 21, 26–29]. Unfortunately, this protocol deeply skews viral communities due to strong discrimination against viruses that sediment outside of the typically selected density layer (including most non-tailed bacteriophages) [20, 30–34], and due to capsid weakening of certain viruses [20, 32]. Similarly, the use of chloroform to disrupt bacterial membranes also compromises the physical integrity of enveloped and some naked viruses [20, 35]. The extensive preference for 0.22 over 0.45 μm syringe filtration has been justified by its better performance at removing small bacteria. However, some studies propose that both strategies efficiently reduce bacterial contamination in host-associated samples [35–37], while 0.22 μm filtration diminished viral DNA yields recovered from human faeces by half in comparison to the use of 0.45 μm filters [38]. This may be explained, at least in part, by the filter retention of giant eukaryotic viruses such as those recently found in human samples [39, 40], or large bacteriophages [41].

Sampling protocols and subsequent preservation procedures can also lead to inaccurate or biased conclusions. Indeed, sample preservation buffer, time and temperature drastically affect the amount of virus detected by flow cytometry [42]. Several strategies are alternatively used for the extraction of virions from host tissues, bacterial biofilms or cellular debris, including the application of physical force by sonication, freezing cycles and homogenizers, though their impact on virus misrepresentation has not been investigated in-depth. Procedures for the concentration of viruses such as tangential flow filtration and ultracentrifugation are also critical steps that reduce virus yield by filter clogging [33, 38] or compromise the integrity of some viruses due to the pressure they are subjected to. Furthermore, DNA extraction kits are disturbing sources of DNA contamination in metagenomic studies [43], skewing viral assemblages by eluting small DNA viral genomes better than non-fragmented large DNA viral genomes [7].

Another controversial source of bias is random amplification, a step that is necessary when the amount of viral genetic material is limiting, preventing NGS, as in the case of extreme environments [44] or many human-associated ecosystems [45]. Three random amplification protocols are widely employed: sequence-independent, single-primer amplification (SISPA, originally called random PCR) [46, 47]; linker amplification shotgun libraries (LASL) [48]; and multiple displacement amplification (MDA) [26, 49]. Each method alters the relative abundance of viruses or provides uneven coverage across sequenced genomes. The SISPA method relies on pseudo-degenerate oligonucleotides, with 6–12 random nucleotides at the 3′ end for random priming and ~ 20 nucleotides of defined sequence at the 5′ end. It has been reported that the annealing bias of the constant part of the primer promotes uneven distribution of sequence reads across the target genome and affects the sensitivity of detection for low-abundance viruses [50]. Pooling SISPA products amplified with different primers provides more uniform coverage patterns [51]. LASL protocol adapted to NGS [52] is another PCR-based method claimed to randomly amplify templates with ultralow quantities [27]. However, LASL requires previous genome fragmentation and DNA-size selection, which makes this approach useless when only a few nanograms of template are available [27]. Additionally, it also exhibits the GC-dependent bias inherent to PCR [27, 53] and overlooks ssDNA viruses [34], though this has recently been overcome with a modified LASL procedure that gives reliable estimates of relative abundance of ssDNA viruses [29]. Unlike SISPA and LASL, MDA is not PCR-based; rather, it amplifies DNA under isothermal conditions [54]. MDA relies on random priming of target DNA with endonuclease-resistant random hexanucleotides and the high fidelity and strong strand-displacement capacity of the podovirus φ29 polymerase to amplify DNA templates with ultralow amounts [49]. Although this method provides more uniform coverage profiles throughout human genomes than those obtained by some PCR-based random amplification methods [55, 56], several biases have been associated with this technology, including chimera formation [57], preferential amplification of circular ssDNA genomes [58] and non-uniform amplification of linear dsDNA genomes. This later bias seems stochastic in single-cell genomics [59] but becomes systematic for nanogram levels of template by under-representing local GC-rich regions [60–64], as previously reported for PCR-based amplification protocols [27, 53, 65–68]. MDA bias is more conspicuous in reactions with higher fold amplification, but in general, > 1 ng of template provides bias affecting a low number of loci in the range of threefold misrepresentation [55, 56, 69, 70]. Modified protocols have claimed to reduce bias by combining MDA with microfluidics [64] or by replacing random hexanucleotides with small oligonucleotides synthesised by enzyme DNA primase [71, 72]. This latter approach also ensures zero-background amplification in the absence of DNA input and even-coverage profiles.

In light of the aforementioned examples, it is reasonable to assume that every step in metagenomic studies of viral assemblages represents a potential source of bias. Continuously falling prices of NGS services are promoting a shift in the scientific goals of metagenomics research from descriptive towards quantitative comparisons of communities. Thus, it is essential to assess multiple replicates in order to gain statistical insight and implement optimised protocols that better preserve the original virome composition. Benchmark studies of virus enrichment and random amplification protocols are required to improve our knowledge about the nature and impact of sources of bias.

In this article, we monitored the composition of synthetic communities formed by seven DNA viruses by quantitative real-time PCR (qPCR), and a natural DNA viral assemblage from human saliva by NGS, along with a simple experiment of virus enrichment coupled with three alternative random amplification procedures. This study provides new information about the bias induced by certain protocol steps, finding that regardless of the random amplification strategy chosen, abundance profiles of viruses from different subjects can be clearly distinguished in ordination plots.

## Methods

### Mock and natural viral communities

Synthetic viral assemblages (henceforth referred to as “mock communities”) were prepared in 1× SM buffer (50 mM Tris pH 7.5, 100 mM NaCl, 10 mM MgSO_4_) and consisted of seven DNA viruses chosen for their different genetic and structural features (Table 1): Vaccinia Western Reserve (WR) was purified from a 36% sucrose cushion prepared in Tris-HCl pH 9.0; bacteriophages lambda, φ29 and M13 were purified by isopycnic CsCl density gradient centrifugation twice [73]; Minute Virus of Mice strain p (MVMp) was firstly purified by centrifugation through a 10–40% sucrose gradient and then through a isopycnic CsCl gradient [74]; human adenovirus 5 (AdenoV) was purified by double CsCl gradient centrifugation [75]; and porcine circovirus 2a (PCV2a) derived from the supernatant of an infected cell culture. Aliquots of each viral stock were independently treated with a cocktail of nucleases (250 U/ml DNAse I, 250 U/ml Nuclease S7, and 100 μg/ml RNAse A; Roche) for 30 min at 37 °C to digest unprotected genetic material such as contaminant DNA from the host or viral DNA from partially unassembled viruses. Then, viral DNA protected in capsids or envelopes was extracted and estimated by absolute qPCR (see below). A balanced mixture of 20 ng of nuclease-resistant genetic material from each of these viruses was pooled together to prepare the first mock assemblage of viruses (mock community 1). As a lower-than-expected proportion of vaccinia virus was detected by qPCR in this mock community, a second one (mock community 2) was prepared taking into account the vaccinia measurement and also with a lower proportion of PCV2a (due to stock exhaustion).

**Table 1:** Features of viruses included in viral mock communities

						Theoretical proportion (%)	
Species	Family	Morphology structure	Diameter (nm)	Genome type	Size (kb)	Mock community 1	Mock community 2
Vaccinia Western Reserve (WR)	*Poxviridae*	Enveloped, brick-shaped virion	200 × 250	Linear dsDNA	194.7	14.28	16.65
Lambda phage (lambda)	*Siphoviridae*	Non-enveloped, head-tail structure	60	Linear dsDNA	48.5	14.28	16.65
Human adenovirus 5 (AdenoV)	*Adenoviriade*	Non-enveloped pseudo *T* = 25 capsid	90	Linear dsDNA	35.9	14.28	16.65
φ29 phage (φ29)	*Podoviridae*	Non-enveloped, head-tail structure	54	Linear dsDNA	19.3	14.28	16.65
M13 phage (M13)	*Inoviridae*	Non-enveloped, rod of filaments	7 × 700–2000	Circular ssDNA	6.4	14.28	16.65
Minute Virus of Mice p (MVMp)	*Parvoviridae*	Non-enveloped *T* = 1 capsid	23	Linear ssDNA	5.1	14.28	16.65
Porcine circovirus 2a (PCV2a)	*Circoviridae*	Non-enveloped *T* = 1 capsid	17	Circular ssDNA	1.8	14.28	0.075

Natural viral assemblages were obtained from 2 to 3 ml of non-stimulated, naturally outflowed saliva samples from healthy volunteers after signing an informed consent document. Samples were diluted 1:4 in 1× SM buffer to reduce saliva viscosity and preserved at 4 °C for up to 30 min until processed. Two pools were elaborated with samples from nine (Unamp1) and seven individuals (Unamp2); six individuals contributed to both pools, and samples were collected 1 week apart.

### Purification protocol

Mock viral communities were subjected to two consecutive low-speed centrifugation rounds at 3000×*g* for 10 min and filtered through 0.22 or 0.45 μm filters (Millex-HV Syringe Filter Unit, PVDF, 33 mm diameter, Millipore). Then, samples were centrifuged at 18000×*g* for 16 h through iodixanol cushions (OptiPrep*™*, density gradient medium, Sigma-Aldrich) consisting of layers of 15 and 50% iodixanol prepared in 1× SM buffer. Viral particles were collected from the interphase between the two layers and subsequently treated with a cocktail of nucleases (250 U/ml DNAse I, 250 U/ml Nuclease S7, and 100 μg/ml RNAse A; Roche) to digest unprotected genetic material. Viral DNA was extracted with 200 μg/ml of proteinase K, 0.5% SDS and phenol:chloroform:isoamyl alcohol (25:24:1) and finally concentrated by ethanol and sodium acetate precipitation. The impact of each of these steps on the composition of mock communities 1 and 2 was assessed in experimental duplicates or triplicates, respectively (Additional file 1: Table S1). Viruses from saliva samples were diluted in three volumes of 1× SM buffer, vigorously shaken by vortex and purified following the same protocol described above, but only using 0.45 μm filters at the filtration step.

### Random amplification

Viral DNA purified from mock communities (1 ng) was randomly amplified by SISPA as previously described [76], using 60 pmol of primer K-8N (Additional file 1: Table S1) in two consecutive rounds of Klenow Fragment (3′ to > 5′exo-; NEBiolabs) extension instead of reverse transcription. Viral DNA from Unamp1 saliva (1 ng) was amplified following the same protocol with the following primers: an FR26RV-primer variant with 12 Ns at the 3′ end (FR20RV-12N) was used in the first step of SISPA to improve coverage evenness [51], and an equimolar mixture of FR20RV primers with 0–4 Ns at the 5′ end were used in the PCR amplification step to improve identification of clusters during Illumina sequencing (SISPA1) [77]. In parallel, a similar strategy was followed using two other primer sets: primers K-12N and K, and primers 454-A-12N and 454-A (Additional file 2: Table S2). Finally, DNA fragments with sizes between 500 and 1500 bp were gel-extracted with a QIAquick Gel Extraction kit (Qiagen) according to the manufacturer’s instructions. PCR products obtained with the three aforementioned primer sets were equally mixed to minimise SISPA bias amplification (SISPA2) [51].

We also amplified DNA from viral assemblages by two alternative MDA kits, both based on φ29 polymerase activity, but differing in random priming strategy. The Illustra Ready-To-Go GenomiPhi V2 or V3 DNA Amplification Kits (GE) use random hexamers, whereas the TruePrime™ Single Cell WGA Kit (Sygnis Biothech) uses PrimPol, a primase enzyme that synthesises random short DNA primers. Viral DNA from mock communities (1 ng) was amplified with a GenomiPhi V2 kit for 2.5 h following the manufacturer’s instructions. Viral DNA from saliva samples was amplified with a GenomiPhi V3 kit using different template amounts and amplification times (Additional file 1: Table S1): MDA_G1 from 1 ng and 2.5 h; MDA_G2 from 1 ng and 10 h; MDA_G3 from 10 pg and 3.5 h; and MDA_G4 from 10 pg and 10 h. A TruePrime™ kit was also used to amplify different template amounts of saliva viral DNA following manufacturer’s instructions: MDA_T1 from 1 ng and 2.5 h; MDA_T2 from 10 pg and 3.5 h.

### Quantitative real-time PCR assays

The composition of mock viral communities was assessed by qPCR in technical triplicates. Oligonucleotides were designed using Primer3Plus [78] under default parameters to amplify targeted regions between 80 and 150 bp (Additional file 1: Table S1). Quantification was performed in 384-well plates with final reaction volumes of 10 μl using two alternative protocols: on a CFX384 Touch thermocycler (BioRad) with SsoFast EvaGreen Supermix (BioRad) kit under this temperature protocol: 30 s at 95 °C + (5 s at 95 °C + 5 s at 60 °C) × 40, or on an ABI PRISM 7900HT SDS thermocycler with a QuantiTect SYBR1 Green PCR Kit (Qiagen, Courtaboeuf, France) under this temperature protocol: 15 min at 95 °C + (15 s at 94 °C + 30 s at 60 °C + 30 s at 72 °C) × 40. Absolute quantification of nuclease-resistant viral genomes was performed using serial dilutions of standards with known copy number as measured by Quant*-*iT™ PicoGreen® dsDNA Assay and NanoDrop*™* 1000 (Thermo Scientific)*.* The standards consisted of linearized plasmids containing the PCR-targeted regions, except for the M13 virus, whose standard was a 650 bp PCR product. Standard curves, with a 5–7 log-linear dynamic range, showed *R*^2^ values above 0.996 and calculated PCR efficiencies between 91.21 and 105.82%. Melt curve analysis of products always showed single peaks; Milli-Q water was used as a non-template control, with no amplification detected in all cases. Data from the ABI thermocycler was analysed using SDS 2.4 software (Applied Biosystems). Mean quantification cycles (*C*_*q*_) of each virus in each sample were converted into absolute concentration (viral genomes/ml) by interpolation on the standard curve (linear regression of the log of standard concentration versus *C*_*q*_). Graphics were drawn using ggplot2 package under R 3.2.3 software [79].

### Illumina sequencing

Viral DNA from saliva samples was fragmented to average lengths of 700–1000 bp by sonication with a Biorruptor Plus (Dioganode), agarose gel extracted, and used to prepare NEBNext*®* Ultra™ DNA libraries (NEBiolabs), with ten PCR cycles of amplification in all cases. Sequencing of the 13 viromes was performed in a MiSeq Illumina Sequencer located at the Parque Científico de Madrid (Madrid, Spain) using a MiSeq Reagent Kit v3 for 600 cycles. A total of 20,365,123 paired reads (2 × 300 pb; 12.22 Gbp in total) were obtained with an average of 1,566,548 reads per virome (Additional file 3: Table S3). Sequences were pre-processed before de novo assembly. First, SISPA primers were trimmed using the Biopieces framework [80]. Three overlapping primer substrings of 15 nt were used as queries for the find_adaptor tool, allowing one error for primer identification. Quality filtration was performed with PrinSeq 0.19.3 Lite [81] with the following parameters: -ns_max_p 1 -ns_max_n 3 -trim_ns_left 1 -trim_ns_right 1 -trim_qual_left 20 -trim_qual_right 20 -trim_qual_type mean -trim_qual_window 2 -trim_qual_step 1 -lc_method entropy -lc_threshold 50 -min_qual_mean 20 -min_len 100 -out_format 1. Contaminating sequences were identified and removed from further analyses by Bowtie2 alignments [82] against the human genome (Genome Reference Consortium Human Build 37 (GRCh37)), a vector dataset (UniVec) and the phiX174 genome (NC_001422.1), under default parameters. Finally, prokaryotic DNA contamination was estimated for a subset of 100,000 randomly selected reads (trimmed to 250 bp) from saliva viromes and from six available saliva metagenomes [83] by BLASTn searches (*e* value < 1e^−10^) against Silva-119 database.

### De novo assembly and contig analyses

Subsamples of 500,000 reads were randomly selected from each metagenome using the random_records tool (Biopieces framework) to perform cross-assembly with the SPAdes genome assembler v.3.6.2 [84] and the next kmers lengths: 21, 33, 55, 77, 99 and 127. A total of 2557 cross-contigs larger than 2 kb were obtained when subsamples from Unamp1, MDA_G1–4, MDA_T1–2 and SISPA1–2 were combined. In a second cross-assembly, 4584 contigs larger than 2 kb were assembled from the 13 available viromes. The impact of the amplification strategy on de novo assembly metrics was assessed for 200,000; 600,000; and 1,200,000 randomly selected reads from each virome. The circular nature of the contigs was assessed following two alternative strategies, looking for reads in the metagenomes simultaneously matching the 5′ and 3′ contig ends: Minimus2 with default parameters from the AMOS package v.3.1.0 [85] and a custom script based on two-direction BLASTn comparisons between contig ends and reads. Only alignments with a minimum overlap of 60 nt and no more than three mismatches and two indels were considered. In addition, we accepted the presence of BLAST hits to small circular viral genomes or plasmids as a valid criterion to identify circular viruses. For that, ORFs were extracted with the Prodigal v2.6.3 tool [86] and significant best BLASTx hits (*e* value < 10^−3^) against the GenBank non-redundant 90 (release 220), and viral protein (downloaded from NCBI in August 2017) databases were computed. The best BLASTx hit among ORFs from the same contig was also used for taxonomic assignment.

### Comparisons of contig profiles among viromes

To compute contig abundance and coverage profiles, reads were aligned to cross-contigs using Bowtie2 under strict alignments parameters (--np 0 --n-ceil L,0,0.02 --rdg 0,6 --rfg 0,6 --mp 6,2 --score-min L,0,-0.2), allowing > 96% identity along the total read length. Coverage profiles were extracted with SAMtools mpileup [87], and cross-contig abundance was normalised by dividing the number of aligned reads by contig length (in kb) and per million reads (RPKM). Trifonov linguistic complexity [88] was calculated in windows of 50 nt and steps of 20 nt with a custom script. Preferential binding sites along contigs for the primers used in SISPA were assessed by looking for 8 nt substrings of the last 15 nt at the 3′ end of each primer. Pearson’s correlations were calculated among coverage profiles of each contig using reads from amplified and unamplified viromes and the stats package in R. Coefficients of variation of contig coverage were computed as the standard deviation of coverage at each nucleotide position (excluding the 5% terminal positions) divided by the mean contig coverage. Lorenz curve analysis was addressed as previously described [89]. SISPA primers at the 5′ end of R1-reads were trimmed with Biopieces framework before Bowtie2 alignment to contigs. Those reads trimmed > 35 bps were considered to have primer-dimers. The percentages of reads with primer-dimers were computed in 50 nt windows with a step of 20 nt using SAMtools and a custom script.

Distances among contigs profiles of saliva viromes were calculated using Bray-Curtis dissimilarities and Sørensen indexes, and ordination plots drawn using non-metric multidimensional scaling (NMDS), available in the vegan package in R. Graphs were obtained using graphics, ggplot2 and vegan packages in R. Pearson’s correlations were also computed among contigs profiles of viromes using the stats package in R.

Distribution of homologous reads among inter-subject saliva samples (Sa101, SaC25 and Sa33) and intra-sample (Unamp1, MDA_G4 and MDA_T2) was modelled as follows: two randomly selected subsamples with 10,000 reads from these viromes were BLASTn-compared in only one direction, and the number of queries with significant hits (*e* value < 10^−10^) was computed after excluding those with hits to reads from the same virome. This procedure was repeated across 10,000 iterations, and the Mann-Whitney *U* test was used to compare distributions of homologous reads.

## Results

### Low-speed centrifugation, filtration and random amplification methods alter composition of mock viral communities

To evaluate the potential effect of purification and random amplification methods on metagenomic studies of viruses, we prepared two mock viral communities (Table 1), each composed of seven DNA viruses with different morphologic (icosahedral, filamentous, naked and enveloped viruses with 17–360 nm diameter) and genetic features (circular and linear, ssDNA and dsDNA genomes). We avoided the use of bacteria as indicator of contamination because the protocol included two consecutive low-speed centrifugation steps and 0.45 μm filtration that reduced the number of colonies in pure cultures of *Escherichia coli*, *Staphylococcus aureus* and *Roseobacter litoralis* by at least 7–8 logarithmic units (Additional file 4: Table S4). To prepare mock communities with equal amounts of genetic material from the seven viruses, the number of nuclease-protected genomes in each viral stock was first determined by absolute qPCR. This quantification strategy avoids underestimation of ssDNA viruses, which are poorly detected by staining reagents [30], and overestimation of viruses damaged structurally during stock preservation or purification. The impact of several independent or combined viral enrichment steps and random amplification protocols were also analysed by qPCR.

**Fig. 1:**
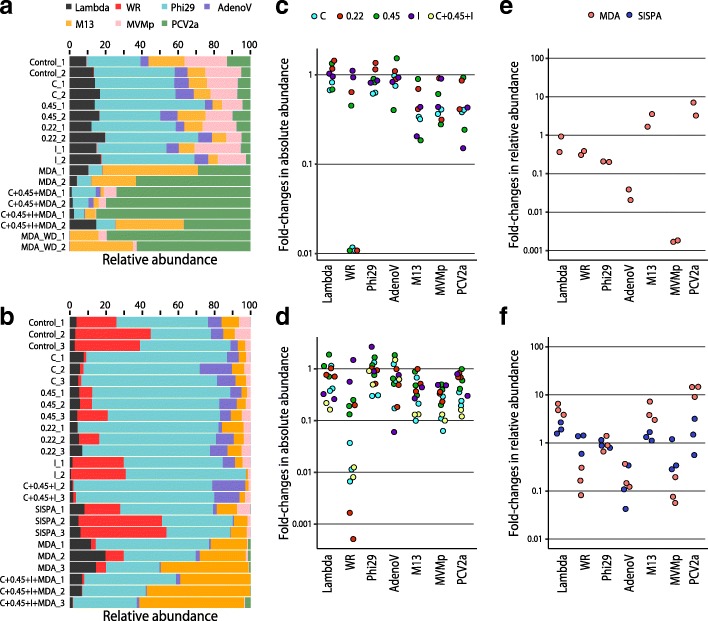
Effects of virus enrichment and random amplification on mock viral communities. Relative proportions of seven DNA viruses (lambda = bacteriophage lambda; WR = vaccinia WR; phi29 = bacteriophage φ29; AdenoV = human adenovirus 5; M13 = bacteriophage M13; MVMp = Minute Virus of Mice p; PCV2a = porcine circovirus 2a) from mock community 1 (**a**) and mock community 2 (**b**) were assessed by qPCR before and after single or combined treatments. Two or three independent replicates were tested for each experiment and noted with numbers 1–3. Sample names include the following identifiers related to treatment: Control, untreated mock assemblage; C, two consecutive centrifugation steps at 3000×*g* for 10 min; 0.45 and 0.22, filter pore size (μm) used during syringe filtration; I, iodixanol cushion; MDA, multiple displacement amplification with GenomiPhi kit; MDA_WD, multiple displacement amplification without denaturation step; SISPA, sequence-independent, single-primer amplification. Fold change in the total amount of each virus genome before and after a given treatment is shown for mock community 1 (**c**) and 2 (**d**). Fold change in relative viral proportion before and after random amplification treatments is shown for mock community 1 (**e**) and 2 (**f**)

Initial proportions of viruses in the mock communities are shown in Fig. 1a, b (controls); most of the viruses were evenly distributed in both experiments. However, mock community 1 showed an unexpectedly low proportion of vaccinia WR genomes compared to the other viruses (0.26% on average), probably due to the decay of viral stability during conservation at 4 °C. Mock community 2 showed under-representation of PCV2a (0.11% in average) due to stock exhaustion during mix preparation (Additional file 5: Table S5).

Low-force centrifugation and filtration reduced the total amount of nuclease-protected viral genomes (Fig. 1c, d). In the case of mock community 2, this reduction was due in part to the 27–150-fold decrease of vaccinia WR genomes after centrifugation and the > 500-fold reduction of the same virus detected in two out the three replicates after 0.22 μm filtration. This negative bias affecting vaccinia caused a drastic reduction in its relative abundance within the mock community (from 22.1–41.8% to 1.4–2.0%) (Fig. 1b). Consistently, vaccinia genomes fell to nearly undetectable levels during centrifugation and filtration of mock community 1 (Fig. 1c). Unexpectedly, small viruses (M13, MVMp and PCV2a) were globally more affected by centrifugation, and to a lesser extent by filtration and iodixanol cushion, than other larger dsDNA viruses (lambda, φ29 and AdenoV). Differences in centrifugation effects between these two groups of viruses across the five independent experimental replicates were statistically significant (*p* = 0.00082, Mann-Whitney Test) but did not notably alter the original assemblage composition, as shown in Fig. 1a, b. In agreement, the combination of these purification steps in mock community 2 reduced the amounts of the small viruses by 6.2–10.0-fold. Iodixanol cushion was the protocol step that best preserved the mock community structure, with a minimal loss of virus particles, which was also true for vaccinia WR virus.

Regarding random amplification, we found that the use of MDA resulted in overrepresentation of small circular ssDNA viruses (M13 or PCV2a), as previously reported [90]. M13 increased its relative abundance by 1.7–3.6 and 3.0–7.2 times in mock communities 1 and 2, respectively, while PCV2a had 3.2–7.1- and 9.1–14.7-fold overrepresentation in mock communities 1 and 2, respectively. As expected, the lack of a denaturation step during the MDA protocol prevented primer annealing to dsDNA molecules, exacerbating the bias towards viruses with circular ssDNA genomes (MDA-WD; Fig. 1a). In contrast, MVMp (and to a lesser extent AdenoV) exhibited consistent decreases (up to ~ 500-fold in the case of MVMp) in relative abundance across the five experimental replicates of MDA random amplification.

Importantly, SISPA amplifications provided less skewed communities, and clearly outperformed MDA in assemblage uniformity, with relative proportions changes in the range of ± 2-fold. The only exception was AdenoV, which was consistently underrepresented in the three experimental replicates by a factor of 2.94-23.6-fold. The loss of AdenoV representation after two alternative random amplification strategies and of MVMp during MDA remains unexplained and deserves further research.

This benchmark study for assessment bias in mock viral communities revealed that both simple viral enrichment and random amplification protocols introduce bias that affects representation.

### Comparison of bias induced by random amplification protocols on human saliva viromes: stochastic bias of MDA amplification from picograms of template and marginal influence of extension times

**Fig. 2:**
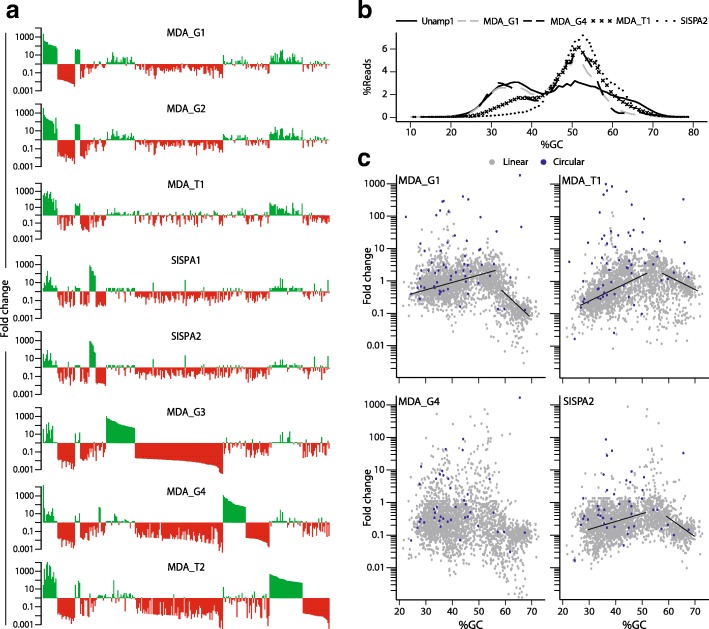
Impact of random amplification bias on reads and contigs from saliva viromes. **a** Fold change of normalised cross-contig abundance (RPKM) between randomly amplified and unamplified viromes are shown. Only those contigs longer than 2 kb with fold change > 50× (green colour) or < 0.02× (red colour) are depicted. Four amplifications were carried out using a GenomiPhi kit with two different DNA template quantities and extension times: 1 ng for 2.5 h (MDA_G1); 1 ng for 10 h (MDA_G2); 10 pg for 3.5 h (MDA_G3); and 10 pg for 10 h (MDA_G4). Amplifications with a TruePrime*™* kit were performed from 1 ng for 2.5 h (MDA_T1) and 10 pg for 3.5 h (MDA_T2). SISPA amplifications were carried out with a single primer (FR26RV-12N; SISPA1) or by pooling the amplification products obtained with three different primers (FR26RV-12N, K-12N, and 454-A-12N; SISPA2). **b** Relative abundance of reads as a function of their average GC content is shown for unamplified and selected randomly amplified viromes. **c** Fold change of 2577 cross-contigs as a function of their average GC content is shown. Small circular cross-contigs are depicted as blue dots and linear cross-contigs as grey dots. Trend lines obtained by linear regression over two different ranges of %GC are shown

To evaluate the impact of random amplification on human viromes, we subjected a pooled-saliva sample to a common viral enrichment protocol and different random DNA amplification strategies. By MiSeq (Illumina) sequencing from the same sample, we obtained nine metagenomes, including an unamplified virome (Unamp1), six viromes obtained after amplification with two commercial MDA kits (GenomiPhi: MDA_G1-4; and TruePrime*™*: MDA_T1-2) and another two with SISPA (SISPA1-2) (Additional file 1: Table S1). Bacterial contamination was detected in our viromes by BLASTn searches against a 16S rDNA database, but the percentage of 16S-related reads was at least ten times lower than in saliva microbiomes from healthy individuals (Additional file 6: Figure S1). De novo cross-assembly of reads from these nine viromes produced 2557 cross-contigs larger than 2 kb, and their normalised abundance was expressed in mapped RPKM. Figure 2a shows the 277 most biased contigs (fold changes > 50 or < 0.02 relative to Unamp1). MDA viromes derived from 1 ng template showed similar patterns of biased contigs, with marginal influence by extension time in GenomiPhi amplification (2.5 and 10 h in MDA_G1 and MDA_G2, respectively) or random priming strategy (GenomiPhi: MDA_G1, and TruePrime*™*: MDA_T1). In contrast, amplification from 10 pg of DNA template notably increased the spectra of contigs affected by bias (MDA_G3, MDA_G4, and MDA_T2) and produced divergent patterns of biased contigs. Therefore, amplifications from low DNA input not only introduce more bias but also increase bias variability. MDA bias patterns differed from those found in SISPA viromes, likely due to fundamental differences between isothermal and PCR-based random amplification methods.

It is well known that MDA based on φ29 polymerase amplifies small plasmids and circular viral genomes more efficiently than linear DNA molecules [90]. As described above for M13 and PCV2a in our mock communities (Fig. 1e, f), the proportion of reads in contigs assigned to circular ssDNA viruses such as inoviruses, microviruses and circoviruses increased in viromes amplified with MDA from 1 ng of template. This over-amplification was exacerbated when using 10 pg (Additional file 7: Figure S2). Furthermore, 10/15 MDA_G1 and MDA_G2 contigs with the highest positive fold change corresponded to small contigs with overlapping ends, suggesting their circular nature, and another showed best BLAST hit to a member of *Microviridae* family (which have circular genomes) (Table 2). Systematic bias towards small circular genomes could also explain over-amplification of many contigs in MDA_T1 but failed to explain the highly variable-positive bias observed when 10 pg was used as template (MDA_G3, MDA_G4 and MDA_T2) (Fig. 2c).

**Table 2:** Circular nature of most overrepresented contigs in MDA_G1 and MDA_G2 viromes

			BLASTx best hit			Overlapping ends	
Contig*	Size (bp)	Fold change	Species	Family	*e* value	Custom script	Minimus2
1473	3117	2965	Enterobacteria phage I2–2	*Inoviridae*	1 × 10^−9^	Yes	Yes
917	4832	501	Microviridae Fen7918_21	*Microviridae*	4 × 10^−84^	Yes	Yes
640	6738	356	Microviridae Fen685_11	*Microviridae*	3 × 10^−24^	Yes	Yes
732	5884	277	Microviridae IME-16	*Microviridae*	0	Yes	Yes
1041	4332	253	Microviridae IME-16	*Microviridae*	0	No	No
1084	4182	205	Vibrio phage fs2	*Inoviridae*	2 × 10^−21^	Yes	Yes
45	39,552	168	Dickeya phage Limestone	*Myoviridae*	5 × 10^−43^	No	No
781	5536	153	Ralstonia phage p12J	*Inoviridae*	2 × 10^−12^	Yes	Yes
674	6397	140	Parabacteroides phage YZ-2015a	*Microviridae*	4 × 10^−31^	Yes	Yes
211	18,180	130	Mycobacterium phage DrDrey	*Siphoviridae*	2 × 10^−21^	No	No
218	17,800	114	Bacillus phage AR9	*Myoviridae*	3 × 10^−18^	No	No
1431	3182	86	Porcine stool-associated circular virus 5	*Circoviridae*	7 × 10^−131^	Yes	Yes
413	10,049	55	Enterobacteria phage Min27	*Podoviridae*	1 × 10^−18^	No	No
1465	3125	52	Enterobacteria phage I2–2	*Inoviridae*	6 × 10^−9^	Yes	Yes
977	4555	50	Gokushovirus WZ-2015a	*Microviridae*	3 × 10^−7^	No	Yes

*Only those contigs with > 50× fold change in MDA_G1 and MDA_G2 are shown

### Systematic bias associated with local regions of extreme GC content skewed human virome composition during PCR-based and isothermal random amplification

The influence of GC content on random amplification of human viromes was studied for reads and contigs. The unamplified virome showed two equivalent peaks of read abundance, with average GC content of 36 and 51%, respectively. However, all amplification strategies produced viromes with a higher number of reads accumulated at the second peak, revealing a systematic positive bias towards reads with average %GC in the range of 44–58 (Fig. 2b). Conversely, GenomiPhi amplification protocols promoted under-representation of reads with average GC content > 60%, while MDA_T1 amplification underrepresented reads with average GC values < 40%, a negative bias that was exacerbated in SISPA-amplified viromes. The 2557 cross-contigs perfectly reproduced the patterns of over- and under-representation described for reads (Fig. 2c). Linear regression analyses of fold changes and average GC content over a range of 30–55% for non-circular contigs showed steeper slopes in MDA_T1 and SISPA2 than in MDA_G1 viromes, whereas the opposite was observed for the GC content range from 60 to 70% (Fig. 2b).

MDA_T1, followed by MDA_G1, showed the lowest number of contigs with > 10 or < 0.1-fold changes relative to Unamp1 (6.2 and 7.6%, respectively). These percentages of highly biased contigs fell to 4.5 and 6%, respectively, when only non-small circular contigs within the 35–65% GC range were analysed. As expected, the proportion of highly biased contigs of SISPA viromes was insensitive to the removal of small circular contigs but exhibited a reduction similar to MDA viromes when those with extreme average GC content were not considered. MDA-amplified viromes from 10 pg of template showed ~ 30% of highly biased contigs which, in agreement with the stochastic bias proposed above, and were insensitive to the removal of small circular contigs or those with extreme GC content. (Additional file 8: Table S6).

### Random amplification under isothermal conditions outperforms PCR-based amplification in coverage uniformity

**Fig. 3:**
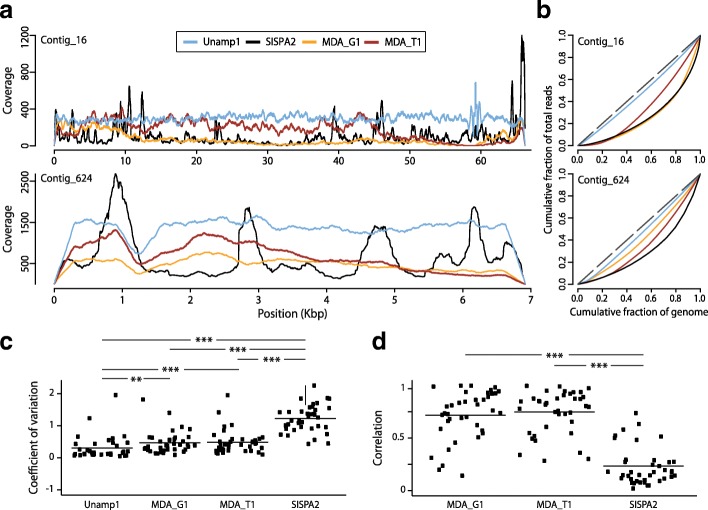
Evenness of contig coverage in saliva viromes obtained by different amplification methods. **a** Coverage profiles across the whole length of two of the most abundant cross-contigs. **b** Homogeneity of read distribution across contig positions is displayed by Lorenz curves. Dashed line depicts the perfect theoretical curve. **c** Coefficients of coverage variation for the 38 most abundant cross-contigs that shared > 50× coverage among analysed viromes. **d** Pearson’s correlations among coverage profiles of amplified and unamplified viromes for the same set of cross-contigs; *p < 0.01; **p < 0.005; and ***p < 0.001

Coverage profiles were inspected across the 38 most abundant contigs, with coverages > 50× in the unamplified virome (Unamp1) as well as in those viromes amplified from 1 ng of the same DNA template (Fig. 3). As exemplified by contig_16 and contig_624, MDA provided more uniform distribution of reads across contigs than SISPA, which agrees with the multiple high-coverage peaks found in previously reported SISPA-amplified viromes [51, 91]. However, coverage profiles in MDA-amplified viromes also showed low coverage in regions where unamplified viromes exhibit even profiles. To further analyse coverage evenness, we drew Lorenz curves by plotting the cumulative fraction of the contig covered by increasing read proportions (Fig. 3b). As expected, curves with the smallest difference from the theoretical even distribution corresponded to the unamplified virome followed by MDA, and SISPA viromes, in this order. To quantify coverage evenness over a representative number of contigs, we calculated coefficients of coverage variation for all 38 inspected contigs (Fig. 3c). By this approach, the highest coefficients of variation corresponded to contigs from the SISPA2 virome, which had average values above 1, and differences from other viromes were statistically significant (*p* < 4.9 × 10^−12^; Mann-Whitney two-tailed tests). Differences between contigs from MDA-amplified and unamplified viromes were also statistically significant (*p* = 0.002 for MDA_G1 vs. unamplified and *p* = 0.0009 for MDA_T1 vs. unamplified), but their average coefficients of variation (under 0.5) were similar to the unamplified virome (0.3). Consistently, Pearson’s correlation values between coverage profiles of amplified and unamplified contigs (Fig. 3d) were lower for SISPA2 than for MDA, and these differences were statistically significant in Mann-Whitney two-tailed tests (*p* < 6.4 × 10^−13^).

In addition, SISPA viromes showed the lowest number of mapped cross-contigs (Additional file 9: Figure S3) and the worst assembly metrics in independent de novo assembly at several sequencing depths (Additional file 10: Figure S4). All together, these results demonstrate a better performance of MDA over SISPA in terms of genome coverage evenness and assembly metrics.

### Coverage unevenness induced by SISPA is partially explained by peaks of high coverage in DNA stretches of low sequence linguistic complexity

**Fig. 4:**
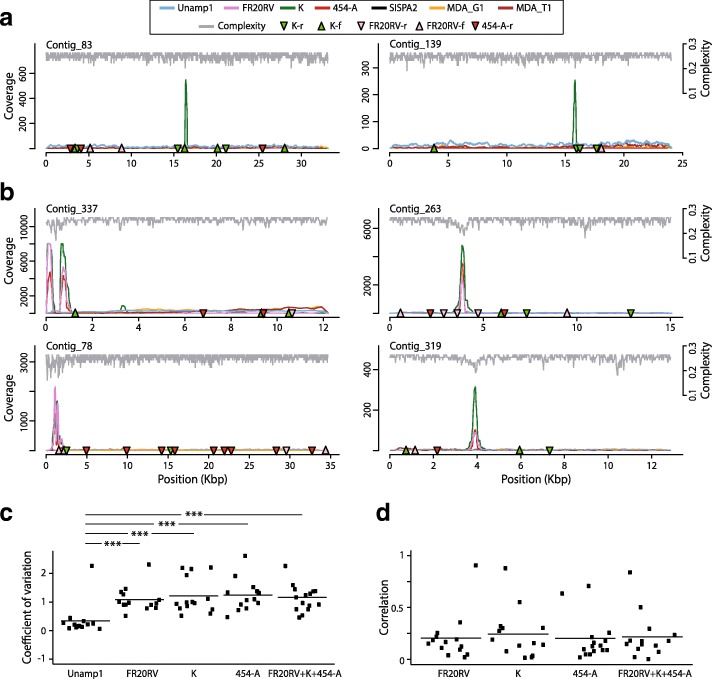
Evenness of contig coverage in saliva viromes obtained by SISPA. **a** Two representative cross-contigs with high-coverage peaks surrounded by sequences with similarity to the constant part of the primers (coloured triangles) used during SISPA. **b** Four representative cross-contigs from SISPA viromes with high-coverage peaks in regions with low linguistic sequence complexity. **c** Coefficients of coverage variation for the 14 most abundant contigs sharing > 50× average coverage among analysed viromes. **d** Pearson’s correlations among cross-contig coverage profiles of unamplified and de-multiplexed SISPA-amplified viromes; *p < 0.01; **p < 0.005; and ***p < 0.001

SISPA bias in coverage evenness has been previously ascribed to preferential annealing of the constant 5′ end of the oligonucleotide. Therefore, pooling of primers has been proposed as a strategy to mitigate bias [51]. Accordingly, around 20% of high-coverage peaks were primer-specific, and some of them were surrounded by sequences with identity to the conserved region of the primer employed (Fig. 4a). However, many other high-coverage peaks were simultaneously obtained by at least two of the three primers used in the SISPA2 virome, suggesting the existence of an alternative source of bias. In agreement, no statistically significant differences were achieved between coefficients of coverage variation for the most abundant contigs mapped with SISPA reads obtained by a single or pool of three primers (Fig. 4c). Moreover, pooling three oligonucleotides failed to improve the correlation index between coverage profiles of SISPA and the unamplified viromes (Fig. 4d). Importantly, we report here that SISPA-induced coverage unevenness is caused, at least in part, by high-coverage peaks in stretches of DNA with low linguistic sequence complexity. Around 30% of these abrupt changes in coverage were not primer-specific but rather corresponded to regions of diminished sequence complexity, as exemplified by contigs shown in Fig. 3b. Regardless of the source of bias, many of the high-coverage peaks were found close to the ends of the contigs, suggesting that these peaks of coverage can also hinder de novo assembly as previously reported [92].

These results indicate that SISPA bias is the result of the convergence of multiple factors including preferential annealing of the constant part of the primer and favoured PCR amplification of DNA regions with low linguistic nucleotide complexity.

### Minimal impact of random amplification bias on beta diversity studies of saliva viromes

**Fig. 5:**
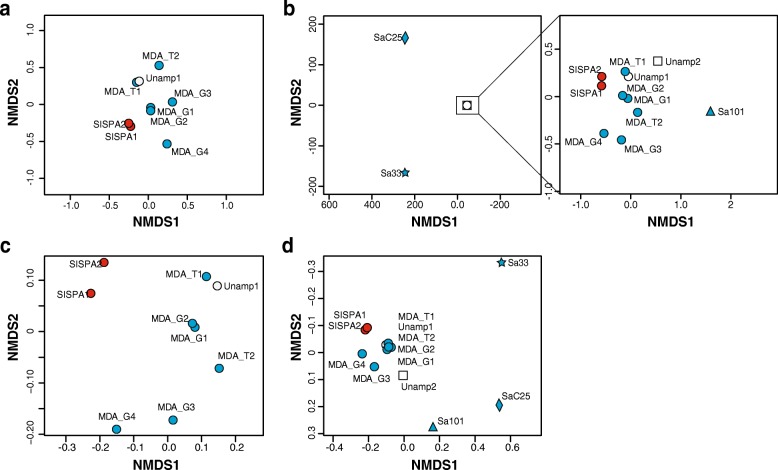
Ordination plots of viromes based on cross-contig abundance profiles. Normalised cross-contig abundances (RPKM) were used to compute Bray-Curtis dissimilarities (**a** and **b**) and Sorensen indexes (**c** and **d**) among viromes. **a** and **c** Dissimilarity matrices of an unamplified virome (Unamp1) and eight derived randomly amplified viromes (MDA_G1-4, MDA_T1-2 and SISPA1-2) was plotted following the NDMS ordination system. **b** and **d** Dissimilarity matrices of the coss-contig obtained with the previous nine viromes, together with two new partially related saliva viromes (Unamp2, an unamplified virome from saliva samples pooled from seven individuals, six of whom also contributed to Unamp1; and Sa101, the GenomiPhi-amplified saliva virome from a single individual who contributed to both Unamp1 and Unamp2 pool samples), and two unrelated samples (GenomiPhi-amplified saliva viromes SaC25 and Sa33 from subjects who did not contribute to either of the pooled viromes tested) was also plotted by NMDS. The NMDS plot at the right of panel **b** represents dissimilarities among all viromes, excluding S25 and S33 (note the differences in the magnitude of the axes). Symbol shape indicates viromes from different samples; white, blue and red colours indicate viromes obtained without random amplification, or randomly amplified by MDA or SISPA, respectively

Random amplification alters the relative abundance of certain members of mock and natural viral assemblages. To assess the impact of this bias at the whole community level, we computed Bray-Curtis dissimilarities among viromes based on cross-contig abundance of normalised mapped reads expressed in RPKMs (Additional file 11: Table S7). These dissimilarities were subsequently drawn in NMDS ordination plots. In agreement with the spectra of the most biased contigs (Fig. 2a), ordination plots showed that viromes obtained after MDA from 1 ng of template localised closer to the unamplified virome than those amplified from 10 pg (Fig. 5a). Furthermore, Pearson’s correlations of 0.69–0.78 were observed between the contig profiles of Unamp1 and those amplified from 1 ng of template, including the SISPA viromes, while correlations with viromes amplified from 10 pg of template ranged from 0.35 to 0.46 (Table 3). Similar results were obtained with the un-weighted Sorensen index (Fig. 5c), which is more sensitive to any bias affecting detection of low abundant individuals. Moreover, nearly the same distribution of viromes was observed with only 200,000 mapping reads (Additional file 9: Figure S3).

**Table 3:** Pearson’s correlations of normalised cross-contig abundances among nine viromes derived from the same saliva sample

	Unamp1	MDA_G1	MDA_G2	MDA_G3	MDA_G4	MDA_T1	MDA_T2	SISPA1
MDA_G1	0.76							
MDA_G2	0.78	0.99						
MDA_G3	0.43	0.63	0.62					
MDA_G4	0.35	0.48	0.52	0.29				
MDA_T1	0.74	0.92	0.91	0.53	0.35			
MDA_T2	0.46	0.45	0.43	0.29	0.23	0.41		
SISPA1	0.72	0.64	0.66	0.36	0.23	0.67	0.28	
SISPA2	0.69	0.61	0.63	0.36	0.21	0.66	0.26	0.99

Importantly, inclusion of two new saliva viromes (SaC25 and Sa33) from subjects that had not contributed to the Unamp1 pool sample in a second cross-assembly (Additional file 12: Table S8) led to a perfect overlap of Unamp1 and all derived viromes obtained after random amplification in a Bray-Curtis-NMDS plot (Fig. 5b). Unamp1 and MDA viromes amplified from 1 ng of template also clustered perfectly in a Sorensen-NMDS plot (Fig. 5d). By contrast, the three unrelated viromes exhibited strong separation in both NMDS plots, with Bray-Curtis dissimilarity and Sorensen index values above 0.98 and 0.65, respectively, and a near absence of Pearson’s correlation (Additional file 13: Table S9–S11), reflecting the uniqueness of human saliva viromes. Moreover, the cluster of Unamp1 and derived viromes in Bray-Curtis NMDS also included two additional MDA-amplified viromes: one from a subject that had been a donor for the Unamp1 pool (Sa101), and another obtained from the pooled saliva of seven individuals (Unamp2), six of whom had also contributed to Unamp1 (Fig. 5b).

Finally, we modelled inter-subject (MDA1-G1, SaC25 and Sa33) distribution of homologous reads (10,000 iterations of BLASTn comparisons between two randomly selected subsamples of 10,000 reads) as an alternative measure of distance. This distribution showed a mean value of 1391.49 ± 86.28 SD homologous reads, which was significantly lower (Mann-Whitney test *p* value < 2.2E−16) than the one similarly obtained for three intra-sample viromes, including Unamp1 and the two most biased viromes (MDA-G4 and MDA_T2; mean value of 4790.81 ± 63.61 SD).

These results suggest that bias induced by isothermal and PCR-based random amplification methods even from picograms of DNA template has a minimal impact on beta diversity studies of saliva viromes from different subjects.

## Discussion

Viruses encompass a wide range of viral morphologies and chemical constituents, which hinders the establishment of universal protocols for purification of viral genomes. However, due to sample limitation and the relatively low amount of viral genetic material in comparison to cellular organisms, the combination of viral enrichment and random amplification protocols is necessary for metagenomic studies of viruses in animal-associated environments. Some protocols for the preparation of human viromes skew the proportions of different viruses [20, 93], hampering efforts to go beyond merely descriptive studies. Some published benchmark studies with mock viral communities have assessed the relative impact of certain sources of bias. However, many of them have employed limited sets of viruses that do not reflect the wide range of morphology, size and genome type found in DNA viruses in nature, or used uneven distributions of viruses that might prevent the identification of some bias sources [18, 32, 33, 37, 94–96]. In our study, we have focused exclusively on DNA viruses because they outnumber RNA viruses in human microbiota. Thus, we have used a mock community composed of seven diverse DNA viruses to explore the bias introduced by simple enrichment protocols (which avoid some of the well-known sources of bias) and several random amplification approaches that can deal with nanograms of input DNA. Preparation of balanced mock viromes must deal with enormous variability, as protocols for stock preparation and storage can lead to non-infectious viral genomes enclosed in partially disrupted capsids or envelopes. We have followed an original approach to prepare balanced mock viral assemblages based on quantification by absolute qPCR after nuclease treatments. This method accurately quantifies viral genomes protected by intact viral particles. The same qPCR method was used to monitor viral gains and losses after four different treatments: low-speed centrifugation, 0.22 and 0.45 μm filtration, and ultracentrifugation through an iodixanol cushion.

The total amount of nuclease-protected vaccinia WR genomes, the largest of the viruses included in our mock communities, was drastically reduced during the two steps aimed at reducing bacterial contamination: 0.22 μm filtration and low-speed centrifugation. The lower impact of 0.45 μm filtration agrees with previous studies that reported the use of 0.45 instead of 0.22 μm filters doubled viral yield [38] and provided a better representation of large viruses such as phycodnavirus, mimivirus and herpesviruses [37, 44, 96, 97]. Regarding bacterial contamination removal, other authors have reported a similar efficiency for 0.22 and 0.45 μm filtration [36]. In our hands, two consecutive low-speed centrifugation steps combined with 0.45 μm syringe filtration reduced the colony-forming units of three pure cultures of bacteria by > 10 million-fold and reduce at least ten times the 16S rDNA content in saliva viromes. Complete physical separation of bacteria and viruses is not possible because of their overlapping sizes, but our protocol provides a good equilibrium between removing most bacteria and including large viruses. A further reduction in centrifugation speed could be explored in future studies to better preserve large viruses such as vaccinia, even more as the increasing output of NGS technologies minimises the negative consequences of tolerating a certain level of bacterial contamination.

The subtle but consistent loss of small viruses observed during the viral enrichment steps was unexpected; further research is necessary to clarify a putative role of aggregation of small viruses under these experimental conditions. Iodixanol treatment was the viral enrichment step that better preserved the community composition, proving to be a reliable strategy of virus concentration. Unlike CsCl density gradients, which efficiently separate virus particles from bacteria but deeply skew viral communities, iodixanol cushions preserve viral communities but fail to exclude bacteria. Since both protocols purify viral particles from low-density material such as free cellular DNA, iodixanol cushions can work synergistically with subsequent nuclease treatments to reduce cellular contamination of viromes.

Stochastic or systematic biases have been associated with all random amplification methods from < 1 ng of template [51, 53, 55, 62, 71, 98]. However, the impact of this bias largely depends on the extent of amplification [99, 100]. In agreement, we found a higher proportion and more divergent pattern of biased contigs in saliva viromes obtained by MDA amplification from 10 pg of DNA template than in those amplified from 1 ng. Therefore, increasing template amount to the nanogram range promotes a shift in the type of bias from stochastic to systematic, reducing dissimilarities with the unamplified virome, as shown in ordination plots. The systematic nature of MDA bias from nanograms of template makes pointless the efforts to reduce bias by pooling independent replicate MDA reactions [101].

Although SISPA and MDA viromes exhibited different patterns of biased contigs, both methods showed similar Pearson’s correlation indexes (0.69–0.78) when compared to the unamplified virome and were located at similar distances in ordination plots. This relatively better performance of MDA in saliva with respect to that observed in mock communities might be explained by the lower proportion small circular genomes in the former, which are usually over-amplified by MDA. In fact, circular contigs from the unamplified saliva virome are only mapped by 0.51% of the total reads.

Positive MDA bias towards small circular viral genomes has been previously quantified as 56× and 212× increases in the relative abundances of two < 2 kb circular viral genomes in soil samples [58], and in 5.7× and 72.6× for two other slightly larger circular ssDNA genomes (5.3 and 6.1 kb) from mock communities [29]. Here, we report a lower MDA over-amplification for the 6.4 kb circular genome of M13 (3.2–7.2×) and for the 1.8 kb circular genome of PCV2a (3.2–14.7×). The higher over-amplification of PCV2a over M13 might correspond to the lower nicking probability of smaller circular molecules. However, an enormous variability in the extent of the bias was observed in small circular contigs from MDA-amplified viromes from the same saliva sample. Since this variability cannot be explained by differences in GC content or contig length, other unknown factors must be participating, such as the stoichiometry between small circular viruses and competing linear templates.

Many of the most over-amplified contigs from MDA saliva viromes corresponded to small circular genomes; however, this source of bias had a minor influence on the global profile of contig abundances, as only two of these contigs were included among the 200 most abundant viruses of the community. Moreover, Bray-Curtis dissimilarities between unamplified and amplified viromes and their relative location in ordination plots remained unaltered after subtraction of small circular contigs. Our study also demonstrated that MDA induces systematic bias against DNA molecules with extremely low and high GC content, and in turn, over-amplification of contigs with average %GC in the range of 45–60%. This type of bias was also identified after SISPA in our studies and has been previously reported for MDA [60–64, 102, 103], LASL [27, 53, 104–106] and in general any method based on PCR amplification [67, 68, 107, 108]. Problems with polymerase accessibility or premature chain termination at the beginning of GC-rich secondary structures have been hypothesised as the most likely cause of their under-representation [60, 66]. Due to the high number of contigs affected, this source of bias might represent the major force that separates unamplified and randomly amplified viromes. Here, we propose that the different ability to deal with regions of high or low GC content might explain the observed differences between SISPA and MDA viromes. Thus, SISPA viromes showed a strong negative bias in reads with %GC between 35 and 40%, while MDA based on random hexamers under-amplified sequences with %GC between 58 and 65%. Interestingly, MDA based on random primers synthesised by DNA primase activity (MGA_T1) outperformed SISPA when dealing with DNA molecules of low GC content, and MDA based on random hexamers when dealing with high GC contigs, as previously reported [71]. These features likely contribute to the nearly perfect overlap of the MDA_T1 and Unamp1 viromes in ordination plots. Picher et al. recently showed no differences between the two alternative priming strategies of MDA in high GC content regions [72]. The discrepancy with our results may be due to the use of different denaturation strategies, MDA kit suppliers of MDA based on random priming or template amounts.

We also identified several biased contigs in saliva viromes that could not be explained by their circular nature or extreme GC content. Similarly, studies on mock communities amplified by MDA revealed a strong negative bias against the ~ 5 kb linear ssDNA genome of MVMp. This genome harbours 43% GC content, excluding any relationship with the previously described negative bias towards GC-rich regions. One possible explanation for under-amplification of small linear templates compared to longer competitors could be a higher impact of progressive template size reduction during MDA.

Coverage evenness has been traditionally used to measure bias induced by random amplification of single genomes. Comparison of three indicators of coverage evenness (Lorenz curves, coefficients of coverage variation and Pearson’s correlation with the unamplified virome coverage) from 38 abundant contigs revealed a better performance for MDA than SISPA. This result was mainly attributable to the presence of multiple peaks of high coverage detected in many contigs from SISPA viromes. Although some of these peaks have been previously ascribed to preferential annealing of the constant part of the pseudo-degenerate primers [50, 109], only a minor proportion of contigs from our saliva viromes harboured primer-specific peaks. Consequently, we failed to improve general parameters of coverage evenness for a set of 14 abundant contigs by pooling three PCR products obtained with alternative primers.

A more detailed inspection of the high-coverage peaks detected by at least two of the primers allowed us to associate many of them with regions of low linguistic sequence complexity. Low-complexity sequences are usually avoided when designing PCR primers [110] or filtered out in BLAST searches [111] in order to prevent unspecific annealing or matching, respectively. These sequences have also been associated with false-positive peak calls due to collapsed repeats in ChIP-s and other sequencing-based functional assays [112]. In our study, we ruled out methodological issues based on mapping or assembling, as high-coverage peaks were obtained by mapping with Bowtie2 under parameters that forced reads to be recruited only once, and detailed inspection of these regions showed no evidence of collapsed repeats. Furthermore, intrinsic issues regarding the low complexity of the template were also excluded because these peaks were absent in the same contigs mapped with reads from MDA or unamplified viromes. Although further research is necessary to understand the molecular basis of this bias towards low linguistic complexity sequences, we hypothesise that preferential annealing of pseudo-degenerate primers to these template regions might be due to the overrepresentation of primers with low sequence complexity. It is well-known that primers with low sequence complexity show favoured stoichiometry for primer-dimer formation, and these dimers might serve as a template during subsequent rounds of PCR amplification, increasing their relative abundance over primers with higher linguistic complexity. Indeed, we show that > 80% of the reads mapping to these high-coverage peaks located in regions with low linguistic complexity contain primer-dimers (Additional file 14: Figure S5). This priming bias, together with the negative bias of genomes with extreme GC content may hamper de novo assembly and contribute to skew the relative contig abundance of SISPA viromes.

Current [27, 64, 70–72, 103, 113] and future efforts to reduce the impact of random amplification bias are desirable and will improve the robustness of longitudinal studies on human viromes. However, our studies suggest that their impact on inter-subject beta diversity may be negligible, due to the well-known uniqueness of human viromes [17, 21, 114]. Our inter-subject saliva viromes showed a significantly lower proportion of homologous reads than those shared by intra-sample viromes, regardless of the random amplification strategy used. This explains why ordination plots based on Bray-Curtis dissimilarities among contig-abundance profiles showed that pooled saliva viromes obtained with or without random amplification perfectly overlapped in a single cluster, separated from two other non-related saliva viromes. Furthermore, this cluster also included the individual saliva virome from a participant from the pooled saliva sample, even though the pool contained equivalent parts of saliva from six other donors. This result agrees with previous studies that showed clustering of saliva viromes from subjects cohabiting in the same household even though only a small proportion of their bacteriophages were shared [22, 28, 115].

## Conclusions

Monitoring balanced mock communities composed of seven different DNA viruses by qPCR revealed that ultracentrifugation through iodixanol cushions, 0.45 μm filtration and random amplification by SISPA preserve the original composition of nuclease-protected viral genomes. By contrast, low-force centrifugation and 0.22 μm filtration led to under-representation of large viruses, and MDA introduced positive bias towards viruses with small circular genomes and negative bias towards small linear genomes.

Comparison of random amplification methods in 13 human saliva viromes (12.22 Gbp) showed that the amplification grade, but not the extension time, was the major source of bias. Thus, stochastic bias observed by amplification from 10 pg of DNA template became systematic when using 1 ng. MDA over-amplification of small circular genomes explains many of the most positively biased contigs but has a minor influence in viral communities dominated by dsDNA bacteriophages such as those found in the oral cavity. In contrast, a negative bias towards DNA sequences with extreme GC content is likely the major force behind isothermal (MDA) and PCR-based (SISPA) systematic bias. MDA priming based on DNA primase activity provided a better representation of contigs with high CG content than that achieved by MDA with random hexamer priming and nearly perfect overlapping with the unamplified virome in ordination plots. SISPA viromes showed uneven coverage profiles with many high-coverage peaks, some of which were primer specific and thus surrounded by sequences with similarity to the constant part of the primer. However, many others were not primer-specific and corresponded to regions of low linguistic sequence complexity.

Amplified and unamplified viromes from the same saliva sample exhibited high proportions of homologous reads and clustered together, separate from unrelated saliva viromes in ordination plots. Therefore, because of the uniqueness of human viromes, random amplification bias has a minimal impact on inter-subject beta diversity studies.

## Additional files

Additional file 1: **Table S1.** Overview of samples and procedures followed for the analysis of mock communities and saliva samples. (XLSX 11 kb)

Additional file 2: **Table S2.** Oligonucleotides used in this study. (XLSX 10 kb)

Additional file 3: **Table S3.** Quality-filtered reads obtained by Miseq-Illumina sequencing and mapped to cross-contigs. (XLSX 9 kb)

Additional file 4: **Table S4.** Effect of several treatments in bacteria removal. (XLSX 9 kb)

Additional file 5: **Table S5.** Virus enrichment and random DNA amplification effects on mock viral communities. Number of genomes determined by absolute qPCR. (XLSX 14 kb)

Additional file 6: **Figure S1.** Percentage of 16S rDNA reads in a set of saliva microbiomes and viromes. (PDF 182 kb)

Additional file 7: **Figure S2.** Taxonomic profile of saliva viromes. (PDF 573 kb)

Additional file 8: **Table S6.** Proportion of highly biased contigs (fold changes > 10× or < 0.1×). (XLSX 9 kb)

Additional file 9: **Figure S3.** Impact of random amplification on beta diversity studies of saliva viromes at different sequencing depths. (PDF 359 kb)

Additional file 10: **Figure S4.** Impact of random amplification and sequencing depth on de novo assembly metrics. (PDF 304 kb)

Additional file 11: **Table S7.** Normalized abundances (RPKMs) of 2570 cross-contigs from Unamp1-derived samples. (XLSX 256 kb)

Additional file 12: **Table S8.** Normalized abundances (RPKMs) of 4598 cross-contigs from Unamp1-derived viromes, Unamp2, and three MDA amplified saliva viromes from different subjects. (XLSX 516 kb)

Additional file 13: **Table S9.** Bray-Curtis dissimilarities among saliva viromes. **Table S10.** Sørensen indexes among saliva viromes. **Table S11.** Pearson’s correlations of normalised cross-contig abundances among nine viromes derived from the same saliva sample and four additional saliva viromes. (XLSX 200 kb)

Additional file 14: **Figure S5.** Profile of reads with primer-dimers in contigs with high coverage peaks at regions of low linguistic complexity. (PDF 429 kb)

## Abbreviations

DTT: Dithiothreitol
LASL: Linker amplification shotgun libraries
MDA: Multiple displacement amplification
MVMp: Minute Virus of Mice strain p
NGS: Next-generation sequencing
NMDS: Non-metric multidimensional scaling
PCV2a: Porcine circovirus 2a
qPCR: Quantitative real-time PCR
RPKM: Reads per contig length in kilobases and per million of reads
SD: Standard deviation
SISPA: Sequence-independent, single-primer amplification
VLPs: Virus-like particles
WR: Vaccinia Western Reserve
