# Supplementary material for: Evaluation of bias induced by viral enrichment and random amplification protocols in metagenomic surveys of saliva DNA viruses
Source: Microbiome. 2018 Jun 28;6:119. doi: 10.1186/s40168-018-0507-3 (PMC6022446; doi:10.1186/s40168-018-0507-3)
Supplement: Supplementary file 6 — Figure S1. Percentage of 16S rDNA reads in a set of saliva microbiomes and viromes. (PDF 182 kb) [file 40168_2018_507_MOESM6_ESM.pdf]

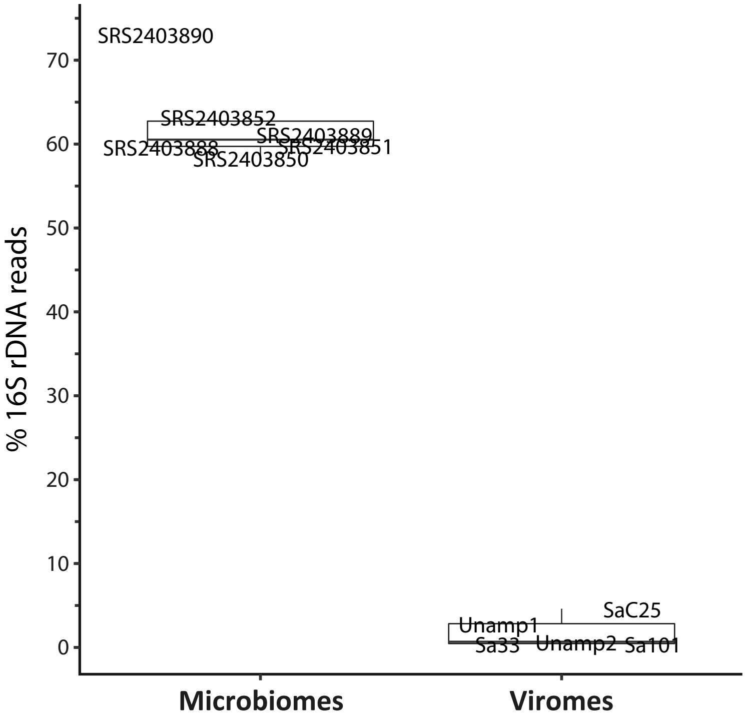

Figure S1. Percentage of 16S rDNA reads in a set of saliva microbiomes and viromes. Microbiomes were obtained from saliva of six healthy individuals (Belstrøm D et al. 2017. NPJ Biofilms Microbiomes) and the five saliva viromes have been obtained in this study. After human DNA contamination removal, high quality reads trimmed to 250 bp were compared by BLASTn against Silva 16S rDNA database (e-vale < 1e-10).
