# Supplementary material for: Evaluation of bias induced by viral enrichment and random amplification protocols in metagenomic surveys of saliva DNA viruses
Source: Microbiome. 2018 Jun 28;6:119. doi: 10.1186/s40168-018-0507-3 (PMC6022446; doi:10.1186/s40168-018-0507-3)
Supplement: Supplementary file 7 — Figure S2. Taxonomic profile of saliva viromes. (PDF 573 kb) [file 40168_2018_507_MOESM7_ESM.pdf]

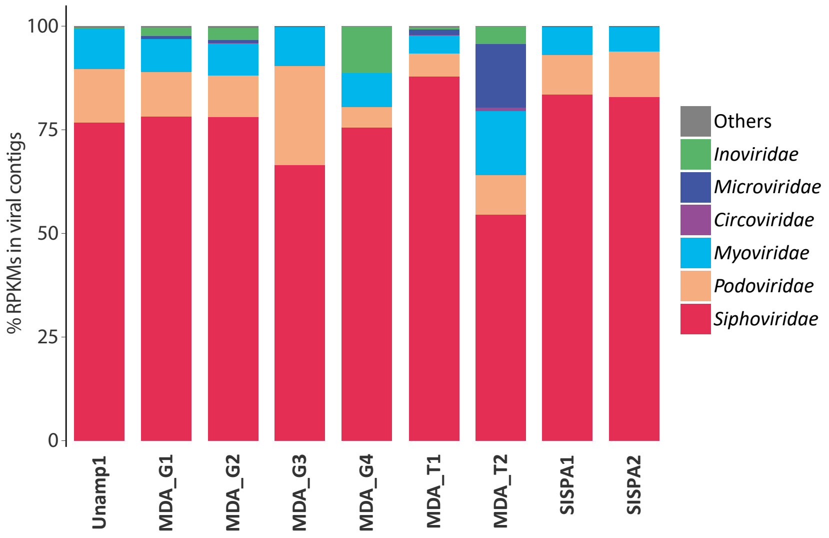

Figure S2. Taxonomic profile of salica viromes. Percentage of RPKMs mapped into contigs assigned to viral families in viral families by BLASTx against a viral protein database. Assignment was based on the best hit found among ORFs of each contig. Less abundant families were grouped into "Others" category.
