# Supplementary material for: Evaluation of bias induced by viral enrichment and random amplification protocols in metagenomic surveys of saliva DNA viruses
Source: Microbiome. 2018 Jun 28;6:119. doi: 10.1186/s40168-018-0507-3 (PMC6022446; doi:10.1186/s40168-018-0507-3)
Supplement: Supplementary file 9 — Figure S3. Impact of random amplification on beta diversity studies of saliva viromes at different sequencing depths. (PDF 359 kb) [file 40168_2018_507_MOESM9_ESM.pdf]

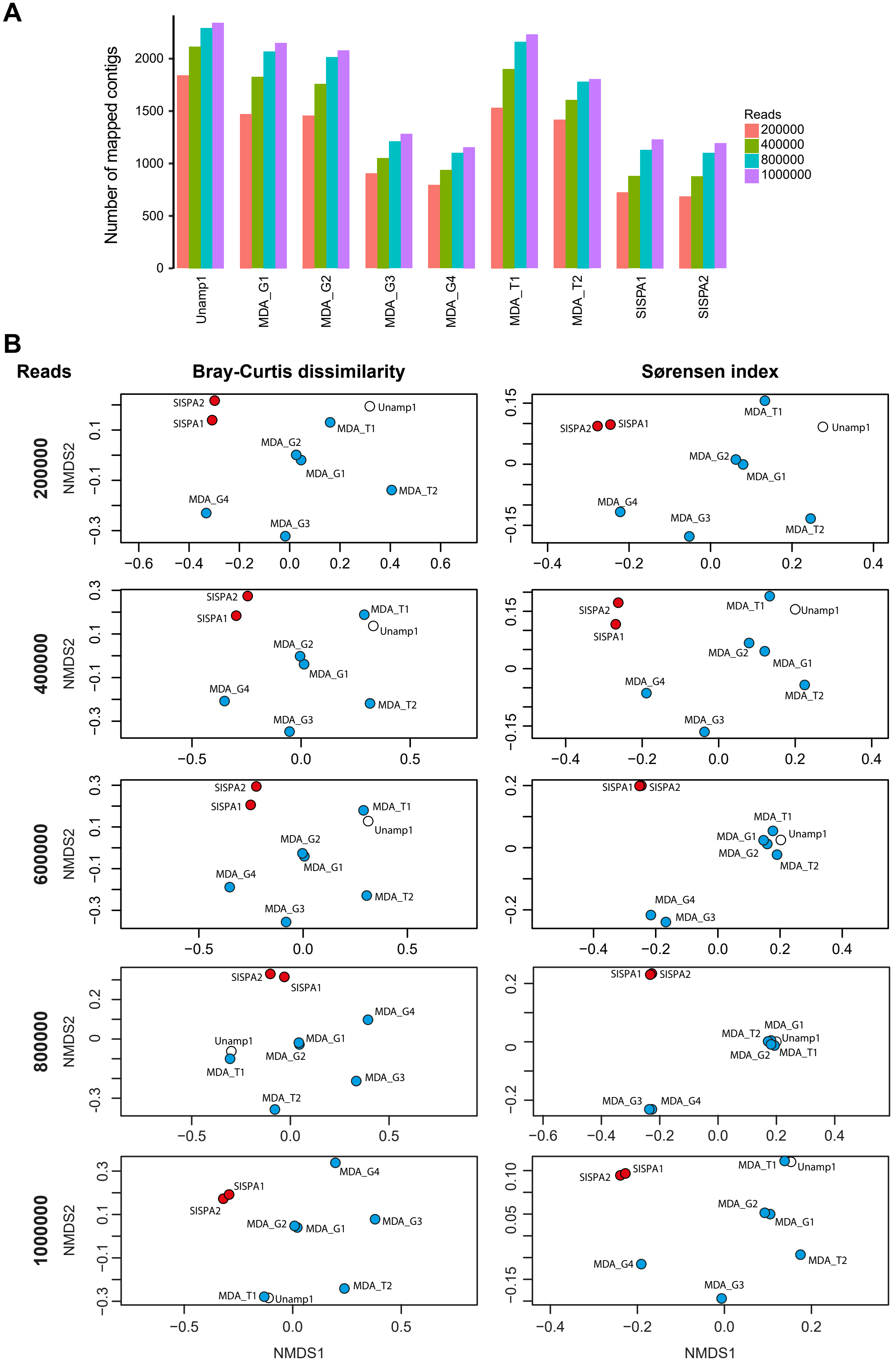

Figure S3. Impact of random amplification on beta diversity studies of saliva viromes at different sequencing depths. (A) Cross-contigs mapped with 200000, 400000, 800000 and 1000000 reads from unamplified and amplified viromes are shown. **(B)** NMDS ordination plots of Bray-Curtis dissimilarities and Sørensen indexes calculated from normalized (RPKMs) cross-contig abundance are shown.
