# Supplementary material for: Evaluation of bias induced by viral enrichment and random amplification protocols in metagenomic surveys of saliva DNA viruses
Source: Microbiome. 2018 Jun 28;6:119. doi: 10.1186/s40168-018-0507-3 (PMC6022446; doi:10.1186/s40168-018-0507-3)
Supplement: Supplementary file 10 — Figure S4. Impact of random amplification and sequencing depth on de novo assembly metrics. (PDF 304 kb) [file 40168_2018_507_MOESM10_ESM.pdf]

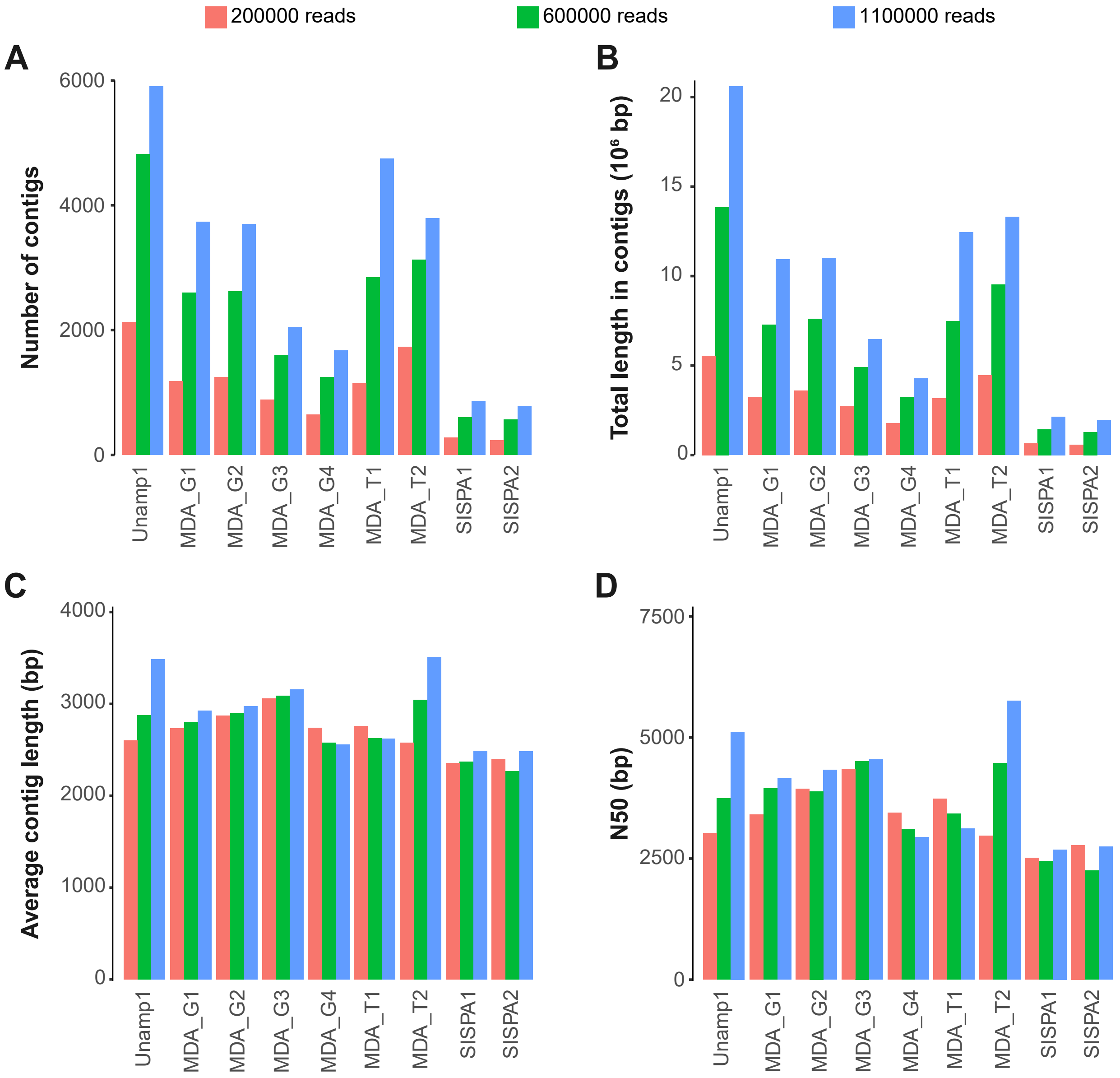

Figure S4. Impact of random amplification and sequencing depth on de novo assembly metrics. (A) Number of contigs, **(B)** total length in contigs, **(C)** average contig length and **(D)** N50, obtained with 200000, 600000, and 1100000 reads for each virome are shown.
