# Supplementary material for: Evaluation of bias induced by viral enrichment and random amplification protocols in metagenomic surveys of saliva DNA viruses
Source: Microbiome. 2018 Jun 28;6:119. doi: 10.1186/s40168-018-0507-3 (PMC6022446; doi:10.1186/s40168-018-0507-3)
Supplement: Supplementary file 14 — Figure S5. Profile of reads with primer-dimers in contigs with high coverage peaks at regions of low linguistic complexity. (PDF 429 kb) [file 40168_2018_507_MOESM14_ESM.pdf]

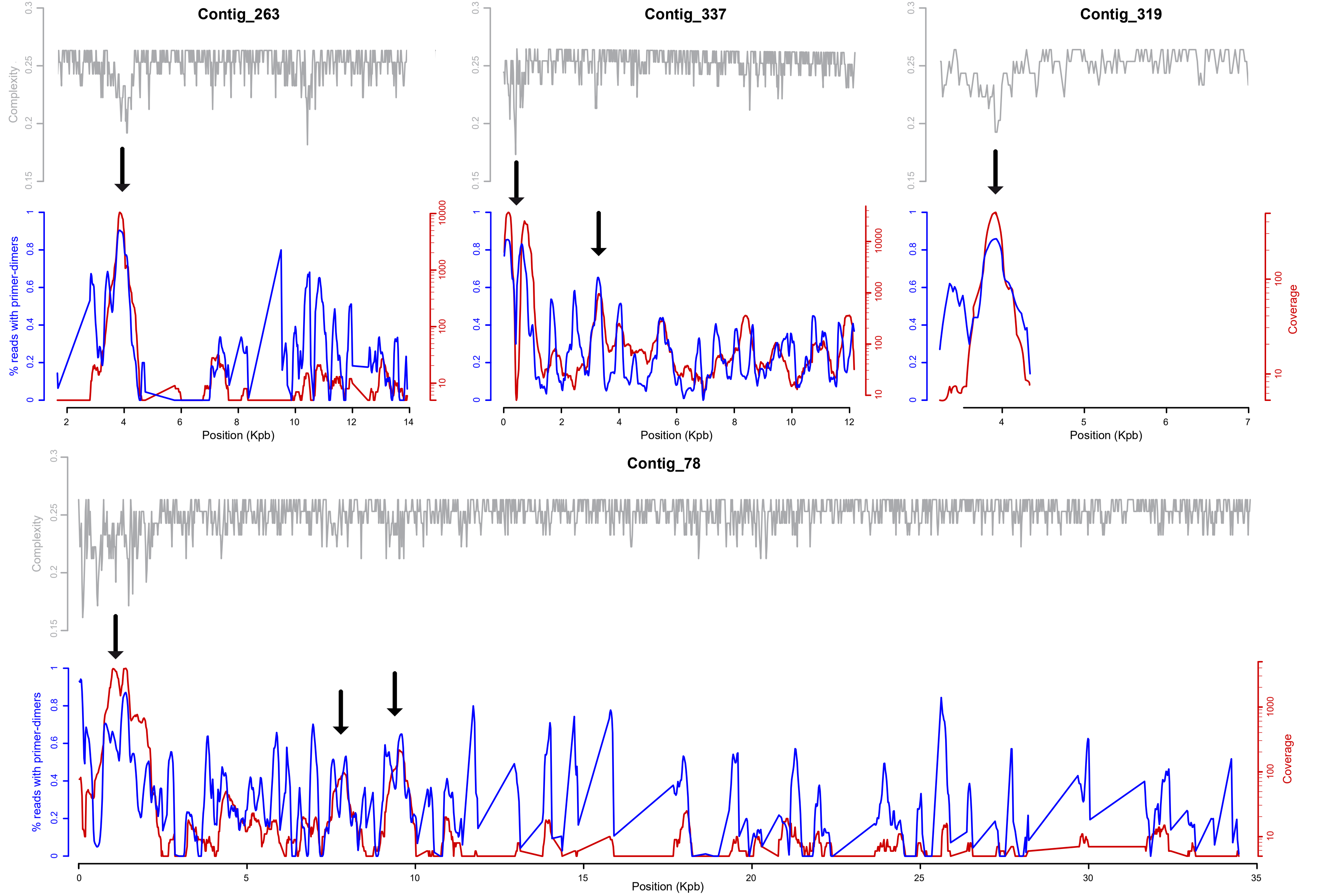

Figure S5. Profile of reads with primer-dimers in contigs with high coverage peaks at regions of low linguistic complexity. Sequences of primers used during SISPA were trimmed at the 5´-end of R1 reads before mapping to the contigs. Percentage of reads trimmed >=35 bp calculated along 50 nt windows with steps of 20 nt are depicted with a blue colour line. Only windows with at least > 5x coverage are evaluated. Trifonov linguistic complexity (grey colour line) and coverage (red colour line) profiles are also shown. Black arrows point to coverage peaks that overlap with low sequence complexity and high % of reads with primer-dimers.
